# Integrative network toxicology and bioinformatics reveal the potential toxicological effects of aspartame on hyperuricemia

**DOI:** 10.1515/med-2026-1557

**Published:** 2026-11-12

**Authors:** Yi Li, Jing Lv, Tiantian Xiao, Haiying Chang

**Affiliations:** Department of Endocrinology, Ganzhou Hospital-Nanfang Hospital, Southern Medical University(Ganzhou People’s Hospital), Ganzhou City, Jiangxi Province, China; Department of Rheumatology, Ganzhou Hospital-Nanfang Hospital, Southern Medical University(Ganzhou People’s Hospital), Ganzhou City, Jiangxi Province, China

**Keywords:** aspartame, hyperuricemia, toxicology, bioinformatics

## Abstract

**Objectives:**

To prioritize molecular links between aspartame-associated records and hyperuricemia-related biology by integrating network toxicology, human transcriptomics, and sensitivity analyses.

**Methods:**

ADMET properties of intact aspartame were predicted, followed by exploratory docking. Records from SwissTargetPrediction, ChEMBL, and CTD were integrated with hyperuricemia-associated genes from GeneCards and OMIM for network and enrichment analyses. GSE160170 provided disease-side transcriptomic evaluation. Sensitivity analyses excluded CTD-only genes and applied graded GeneCards filters.

**Results:**

Intact aspartame showed low predicted permeability and high model-predicted alerts for genotoxicity, hepatotoxicity, nephrotoxicity, and neurotoxicity. The primary analysis identified 308 candidate records, 59 overlapping genes, and five hub candidates: IL1B, IL6, PPARG, INS, and TNF. GSE160170 showed upregulation of IL1B, IL6, TNF, PTGS2, and VEGFA and downregulation of MME. Removing 171 CTD-only candidates reduced the overlap to 22 genes and retained only IL1B among the five hubs, while several inflammatory pathways remained significant. GeneCards filtering narrowed the network but preserved disease-side hub ranking. Docking suggested modest-to-weak structural compatibility.

**Conclusions:**

The combined evidence prioritizes an IL1B-centered inflammatory network as the most reproducible signal. IL6 and TNF had disease-side support but CTD-dependent exposure-side inclusion; PPARG and INS remained exploratory. The study does not establish an aspartame-specific causal effect on serum urate.

## Introduction

Hyperuricemia, defined by sustained elevation of serum urate, is the biochemical prerequisite for gout and a common metabolic disorder [1], 2]. Serum urate reflects the balance between production during purine catabolism and renal and intestinal excretion [3], [4], [5], [6], [7]. Persistent imbalance promotes monosodium urate crystal deposition and is associated with renal injury, endothelial dysfunction, oxidative stress, and cardiometabolic disease [3], [8], [9], [10], [11]. Once deposited, urate crystals activate innate immune pathways – most notably the NLRP3 inflammasome–IL-1β axis – and drive acute and chronic gouty inflammation [12], [13], [14], [15], [16]. Hyperuricemia therefore lies at the intersection of urate handling, metabolic regulation, and inflammation. Although its core production and transport mechanisms are well characterized, the dietary and environmental exposures that may modify these processes remain less clearly defined.

Aspartame (L-aspartyl-L-phenylalanine methyl ester) is a widely used high-intensity sweetener in beverages, foods, and pharmaceutical formulations. After ingestion, it is rapidly hydrolyzed in the gastrointestinal tract to phenylalanine, aspartic acid, and methanol; unchanged aspartame is not considered a major systemic exposure species [17], [18], [19]. This metabolic fate is important because responses associated with consumption may arise from the metabolites, downstream exposure effects, or the parent compound under local or experimental conditions. Regulatory and toxicological assessments have focused largely on safety at permitted intake levels, yet whether exposure intersects with metabolic and inflammatory pathways in susceptible individuals remains of interest [17], 18]. Recent network-based studies have examined potential associations between aspartame and several complex diseases [20], [21], [22], but its relationship to hyperuricemia has not been systematically evaluated. It is therefore unclear whether aspartame-associated records converge on the machinery that controls serum urate or primarily on the inflammatory and metabolic processes accompanying hyperuricemia and gout.

That distinction is biologically important. Serum urate is governed directly by purine-degradation enzymes and renal or intestinal transporters, whereas cytokines such as IL-1β, IL-6, and TNF mainly amplify inflammation after urate accumulation and crystal deposition. A systems-level analysis can reveal coherent intersections across metabolic, vascular, renal, and immune pathways, but network overlap alone cannot establish exposure specificity, target engagement, or causality. The exposure databases used for such analyses also represent different evidence types, ranging from computational predictions to target-search records and curated chemical–gene associations. Broad candidate retrieval should therefore be paired with independent disease-side evidence and sensitivity analyses that test the influence of weak disease associations and source-specific exposure records.

We addressed these questions using an evidence-calibrated network toxicology framework. Intact aspartame served as the standardized parent-compound index for ADMET profiling, database retrieval, and exploratory docking, with its rapid presystemic hydrolysis considered explicitly during interpretation. Source-stratified candidate records were integrated with hyperuricemia-associated genes and examined by protein–protein interaction analysis, module detection, topological prioritization, and functional enrichment. GSE160170 provided an independent human transcriptomic context. Complementary sensitivity analyses then assessed dependence on CTD-only exposure records and GeneCards relevance-score thresholds. Finally, the findings were organized by evidence tier and by their relation to direct urate production or transport, indirect renal or metabolic context, and downstream gout inflammation. The aim was to identify experimentally testable priorities, not to infer an aspartame-specific causal mechanism of hyperuricemia.

## Materials and methods

### Physicochemical and ADMET profiling

The chemical structure and canonical SMILES of intact aspartame (PubChem CID 134601) were retrieved from PubChem. The SMILES was submitted to ADMETlab 3.0 on December 10, 2025. Recorded descriptors included molecular weight, octanol–water partition coefficient (LogP), topological polar surface area (TPSA), aqueous solubility (LogS), Caco-2 permeability (LogPapp), blood–brain barrier penetration probability, plasma protein binding, apparent volume of distribution (log VDss), CYP2D6 inhibitor and substrate probabilities, predicted elimination half-life, and model-predicted toxicity endpoints. Results were summarized in R version 4.3.1. Toxicity probabilities were interpreted as QSAR-derived alerts rather than experimental estimates of toxicity or clinical risk.

### Collection and classification of aspartame-associated records

An intentionally broad retrieval strategy was used to minimize omission of potentially relevant records. Because the databases differed in evidence type, source labels were retained throughout the analysis. Search inputs, dates, record counts, and record-level annotations are provided in Supplementary Methods S1 and Supplementary Tables S1A–S1B.

SwissTargetPrediction was queried on December 9, 2025, with the canonical SMILES of aspartame and Homo sapiens selected as the target species. The primary analysis retained all 100 returned labels to maximize sensitivity. The available export did not include prediction probabilities; none were reconstructed retrospectively.

The ChEMBL target-search interface was queried on December 9, 2025, using the term “aspartame.” Of 93 exported rows, 34 were annotated as Homo sapiens. Expansion and deduplication of multi-accession entries yielded 44 unique human UniProt accession/gene labels. Because the export represented a target search rather than aspartame-specific bioactivity data, these entries were classified as database-retrieved human target records; no affinity, assay-confidence, or pChEMBL threshold was applied.

CTD was queried on December 9, 2025, using the specific aspartame entry (Chemical ID D001218). After blank rows were excluded, 178 unique genes remained. Gene ID, Interaction Count, and Organism Count were retained as annotations, with Interaction Count treated as the number of curated records rather than a confidence measure. The gene-level summary lacked study-specific context and parent-versus-metabolite attribution; accordingly, CTD entries were classified as curated aspartame-associated genes rather than direct molecular targets of intact aspartame.

Standardization of gene and protein labels followed by duplicate removal yielded 308 unique candidate records across the three sources.

### Collection of hyperuricemia-associated genes

GeneCards and OMIM were queried with the term “Hyperuricemia.” GeneCards returned 1,455 genes, and their relevance scores were retained as database-specific annotations. The OMIM export contained 111 records representing 110 unique gene or locus labels. Thirty-seven labels occurred in both sources, leaving 1,528 unique hyperuricemia-associated labels after integration. The unfiltered set was used in the primary analysis to preserve discovery sensitivity; the effect of GeneCards filtering was examined separately.

### Identification of shared genes and network analysis

Intersection of the 308 candidate records with the 1,528 hyperuricemia-associated labels in OECloud yielded 59 shared genes. These genes were submitted to STRING version 11.5 with the organism restricted to Homo sapiens. At a minimum combined score of 0.400 (medium confidence), the exported network contained 57 nodes and 1,082 interaction records; genes without exported interactions were absent from the edge table. Network visualization and downstream analyses were performed in Cytoscape version 3.9.1.

### Module detection and hub-gene prioritization

The highest-scoring module identified with MCODE was retained for further analysis. Network nodes were ranked with six cytoHubba algorithms: maximal clique centrality, maximum neighborhood component, Degree, edge percolated component, Closeness, and Betweenness. The top 10 genes in each ranking were intersected, yielding IL1B, IL6, PPARG, INS, and TNF. These genes were treated as topological priorities rather than confirmed or aspartame-specific targets.

### Functional enrichment and interaction analysis

Gene Ontology (GO) and Kyoto Encyclopedia of Genes and Genomes (KEGG) enrichment analyses of the 59 shared genes were performed with Hiplot Pro. GO analysis covered biological process, molecular function, and cellular component categories. Nominal p<0.05 and false-discovery rate (FDR) <0.05 were used as significance criteria. Enrichment of the five hub candidates and their functional interaction network were examined exploratorily with Hiplot Pro and GeneMANIA, respectively.

### Exploratory molecular docking

Intact aspartame was used as the standardized parent ligand to maintain consistency with the database workflow; metabolite-specific docking was not performed. Protein structures were obtained from the RCSB Protein Data Bank: IL1B (1L2H, 1.54 Å), IL6 (1ALU, 1.90 Å), PPARG (1ZGY, 1.80 Å), INS (1BEN, 1.40 Å), and TNF (5UUI, 1.40 Å). The available preparation records documented removal of water molecules, addition of polar hydrogens, and conversion of receptor and ligand structures to PDBQT format. Docking was performed with AutoDock Vina version 1.2.2 [23].

The available workflow specified a 30 Å × 30 Å × 30 Å search box and a grid-point distance of 0.05 nm. Exact grid centers, exhaustiveness, random seeds, number of output modes, energy range, and raw Vina logs were not recoverable. Co-crystal ligand redocking, positive-control docking, and RMSD validation were not performed. Docking scores are therefore reported in kcal/mol and interpreted as exploratory estimates of static structural compatibility. Receptor-specific caveats, contact residues, and the full parameter audit are provided in Supplementary Methods S6 and Supplementary Table S8.

### Human transcriptomic evaluation

Disease-side transcriptomic consistency was examined in GSE160170, a human peripheral blood mononuclear cell microarray dataset comprising six patients with primary gout and six healthy controls. The gout group included three intercritical and three acute-gout samples. Normalized log2 expression values were used for quality assessment, principal component analysis, sample-level visualization, and differential-expression analysis. The primary comparison included all gout samples vs. controls and was analyzed with limma in R. Benjamini–Hochberg-adjusted p<0.05 and |log2 fold change| >1 were prespecified as differential-expression criteria.

The five primary hub candidates and the 59 shared genes were evaluated against the available probe-to-gene mapping. IL1B, IL6, and TNF had uniquely mapped representative probes. Reliable probe identities could not be assigned to PPARG or INS from the significant-probe annotation; these genes were therefore classified as not evaluable rather than nonsignificant. Comparisons involving the intercritical and acute-gout subgroups were exploratory because each subgroup contained only three samples. Preprocessing, quality-control, and probe-mapping procedures are detailed in Supplementary Methods S2.

### CTD-only source-deletion sensitivity analysis

Dependence on CTD-derived evidence was assessed by defining CTD-only genes as those present in CTD but absent from both SwissTargetPrediction and ChEMBL. These genes were removed, and the remaining candidate set was re-intersected with the original hyperuricemia-associated set. The STRING network was restricted to retained genes, and degree, betweenness centrality, and closeness centrality were recalculated in NetworkX version 3.6.1.

KEGG over-representation analysis was repeated for the original and CTD-only deletion sets using the same human gene-to-pathway mapping and Benjamini–Hochberg correction. Background definition, pathway testing, network provenance, and gene-level results are reported in Supplementary Methods S3, Supplementary Figure S5, and Supplementary Table S5.

### GeneCards filtering sensitivity analysis

Disease-side robustness was evaluated by retaining all OMIM records and applying four GeneCards relevance-score thresholds: the observed median (0.690937), score ≥1.0, the upper quartile (1.074029), and score ≥2.0. At each threshold, the candidate–disease overlap, restricted STRING network, degree ranking, and KEGG enrichment were recalculated under identical analytical settings. Detailed procedures and complete results are provided in Supplementary Methods S4, Supplementary Figure S6, and Supplementary Table S6.

### Evidence integration and relation to urate biology

Findings were integrated in three evidence tiers. Tier 1 comprised genes or pathways retained after CTD-only deletion and, where evaluable, supported in GSE160170. Tier 2 comprised genes supported in the human dataset but dependent on CTD for exposure-side inclusion. Tier 3 comprised CTD-dependent genes without independent transcriptomic support and pathways not retained after source deletion. A targeted literature-based classification further distinguished direct urate production, core urate transport, indirect renal or metabolic context, and downstream gout inflammation. These categories guided interpretation but did not constitute a quantitative confidence score; full definitions appear in Supplementary Methods S5 and Supplementary Table S7. The workflow of this study is illustrated in Figure 1.

**Figure 1:**
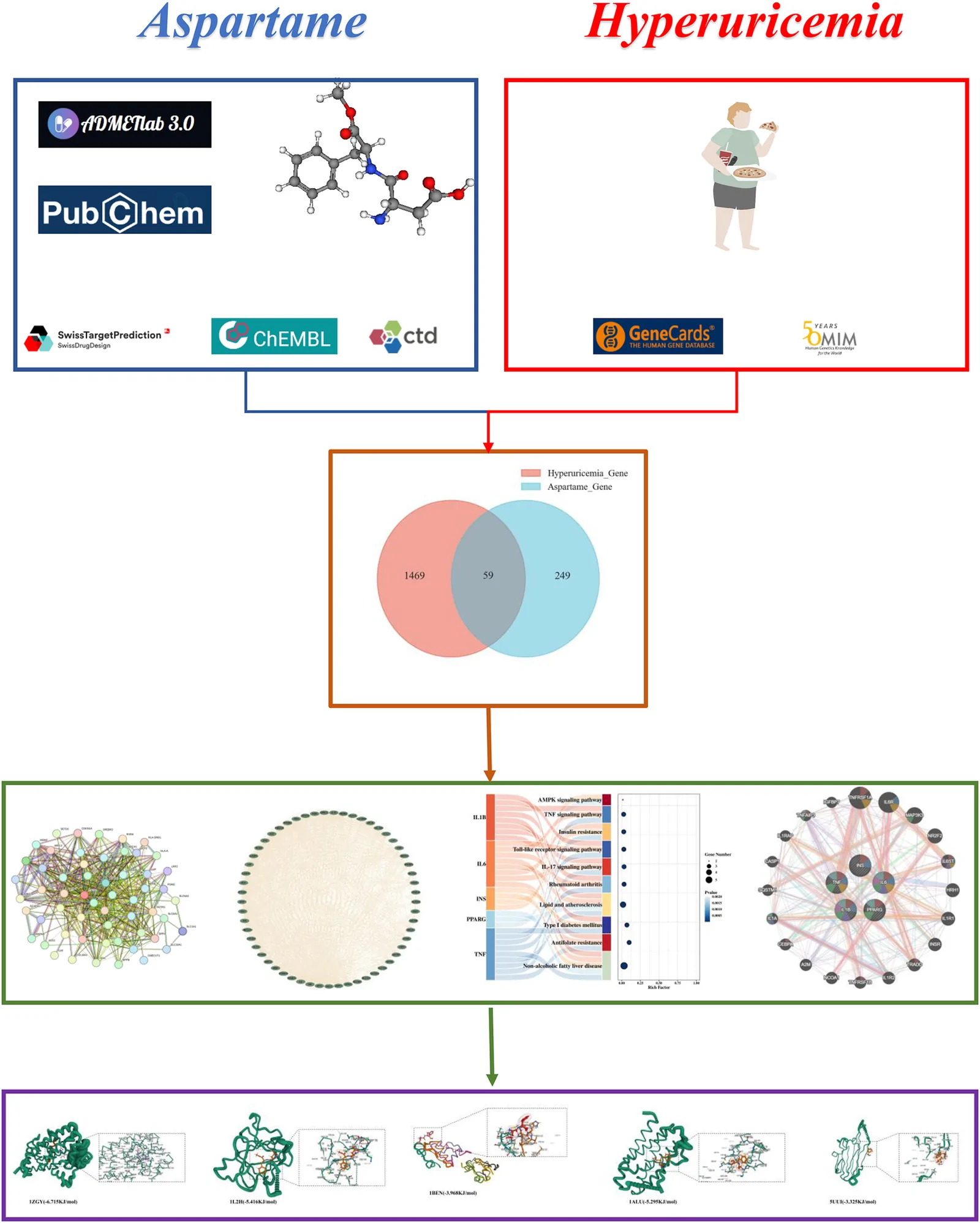
Primary research workflow. The figure depicts the original parent-compound network-toxicology and docking workflow. Independent transcriptomic evaluation and sensitivity analyses are reported in the Supplementary Materials.

## Results

### Chemical characteristics, pharmacokinetic profile, and model-predicted toxicity signals of aspartame

Aspartame is a polar dipeptide methyl ester composed of aspartic acid, phenylalanine, and a methyl ester moiety (Figure 2A). The intact parent molecule had a molecular weight of 294.12 Da, LogP of −1.09, topological polar surface area of 118.72 Å^2^, and predicted LogS of −1.41 (Figure 2B and Table 1). Consistent with this hydrophilic profile, ADMETlab predicted low Caco-2 permeability (LogPapp=−6.28) and a very low probability of blood–brain barrier penetration (0.007). Predicted plasma protein binding was 45.65 %, log VDss was −0.34, and the CYP2D6 inhibitor and substrate probabilities were 0.006 and 0.636, respectively. The predicted elimination half-life was 1.45 h. These estimates describe the intact structure submitted to the model and do not represent measured systemic exposure after ingestion.

**Figure 2:**
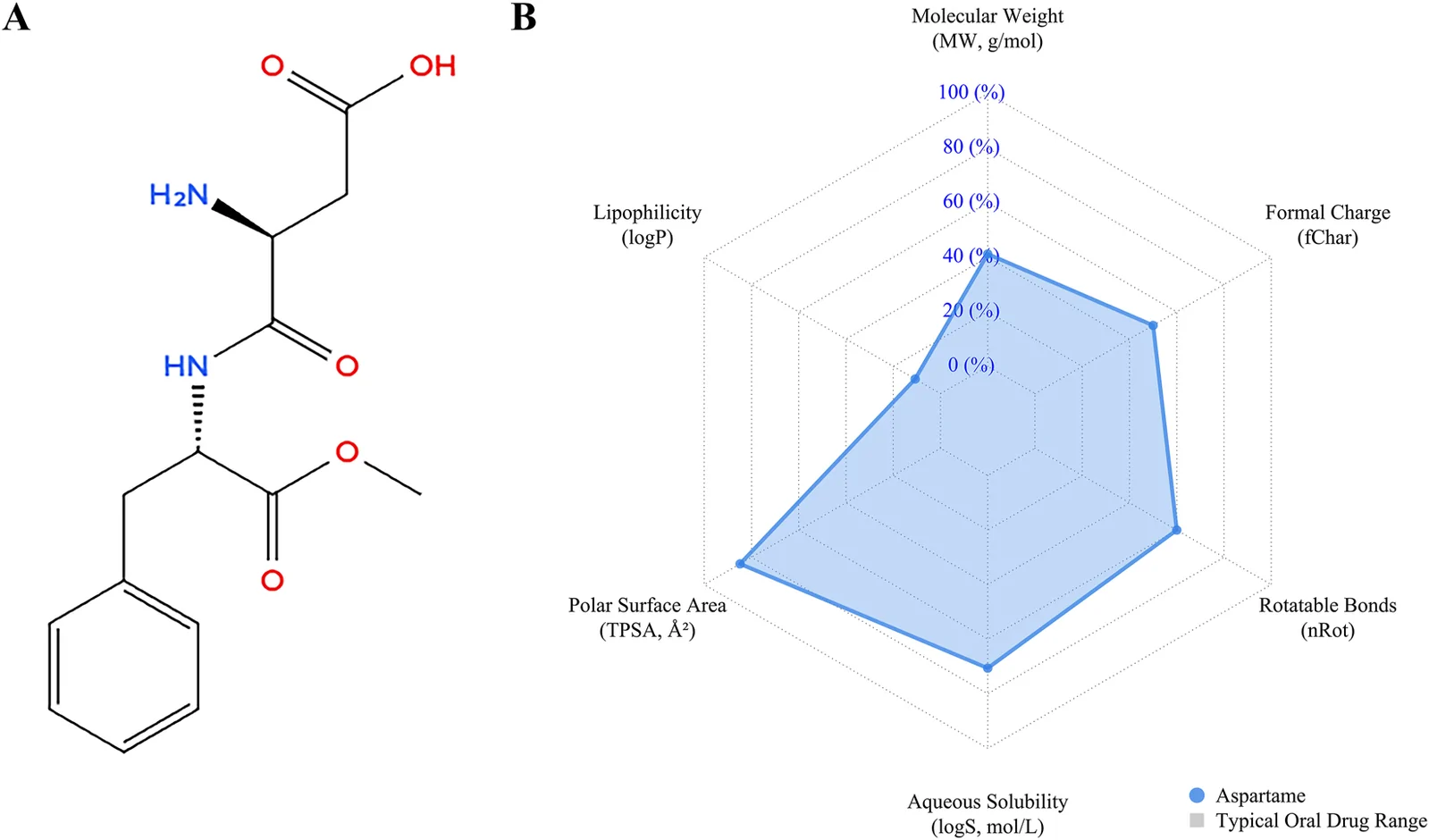
Physicochemical properties of intact aspartame. (A) Chemical structure. (B) Radar plot of selected parent-compound physicochemical descriptors.

**Table 1:** Model-predicted physicochemical and pharmacokinetic properties of intact aspartame.

Parameters	Predicted value	Interpretive note
Molecular weight	294.12 Da	Parent-compound descriptor
LogP	−1.09	Hydrophilic profile
TPSA	118.72 Å^2^	High polar surface area
Aqueous solubility (LogS)	−1.41	Model-predicted solubility
Caco-2 permeability (LogPapp)	−6.28	Low predicted permeability; logarithmic model output
BBB penetration probability	0.007	Very low predicted signal
Plasma protein binding	45.65 %	Model-predicted value
Apparent volume of distribution (log VDss)	−0.34	Log-transformed distribution descriptor
CYP2D6 inhibitor probability	0.006	Very low predicted inhibitor signal
CYP2D6 substrate probability	0.636	Model-predicted substrate signal
Predicted half-life	1.45 h	Not measured exposure kinetics

All values refer to the intact parent structure submitted to ADMETlab and do not represent measured systemic exposure after ingestion. LogPapp and log VDss are logarithmic model outputs.

The toxicity models returned high alert probabilities for genotoxicity/Ames activity (0.998), hepatotoxicity (0.905), nephrotoxicity (0.906), and neurotoxicity (0.936) (Table 2). These values were treated as QSAR-derived signals requiring experimental confirmation, not as observed toxic effects or estimates of clinical risk.

**Table 2:** Model-predicted toxicity alert probabilities for intact aspartame.

Toxicity endpoint	Prediction probability	Interpretation
Genotoxicity	0.998	High model-predicted alert; not experimental genotoxicity
Hepatotoxicity	0.905	High model-predicted alert; not evidence of liver injury
Nephrotoxicity	0.906	High model-predicted alert; not evidence of kidney injury
Neurotoxicity	0.936	High model-predicted alert; not evidence of clinical neurotoxicity

Probabilities are QSAR-derived model outputs and should not be interpreted as observed toxicity, organ injury, or clinical risk.

### Primary network toxicology analysis of aspartame in hyperuricemia

The source-stratified search yielded 100 SwissTargetPrediction labels, 44 ChEMBL-derived human target records, and 178 curated CTD aspartame-associated genes. After standardization and deduplication, 308 unique candidate records remained. In parallel, 1,455 GeneCards records and 110 unique OMIM gene or locus labels produced 1,528 hyperuricemia-associated labels. Their intersection comprised 59 shared genes (Figure 3A **)**.

**Figure 3:**
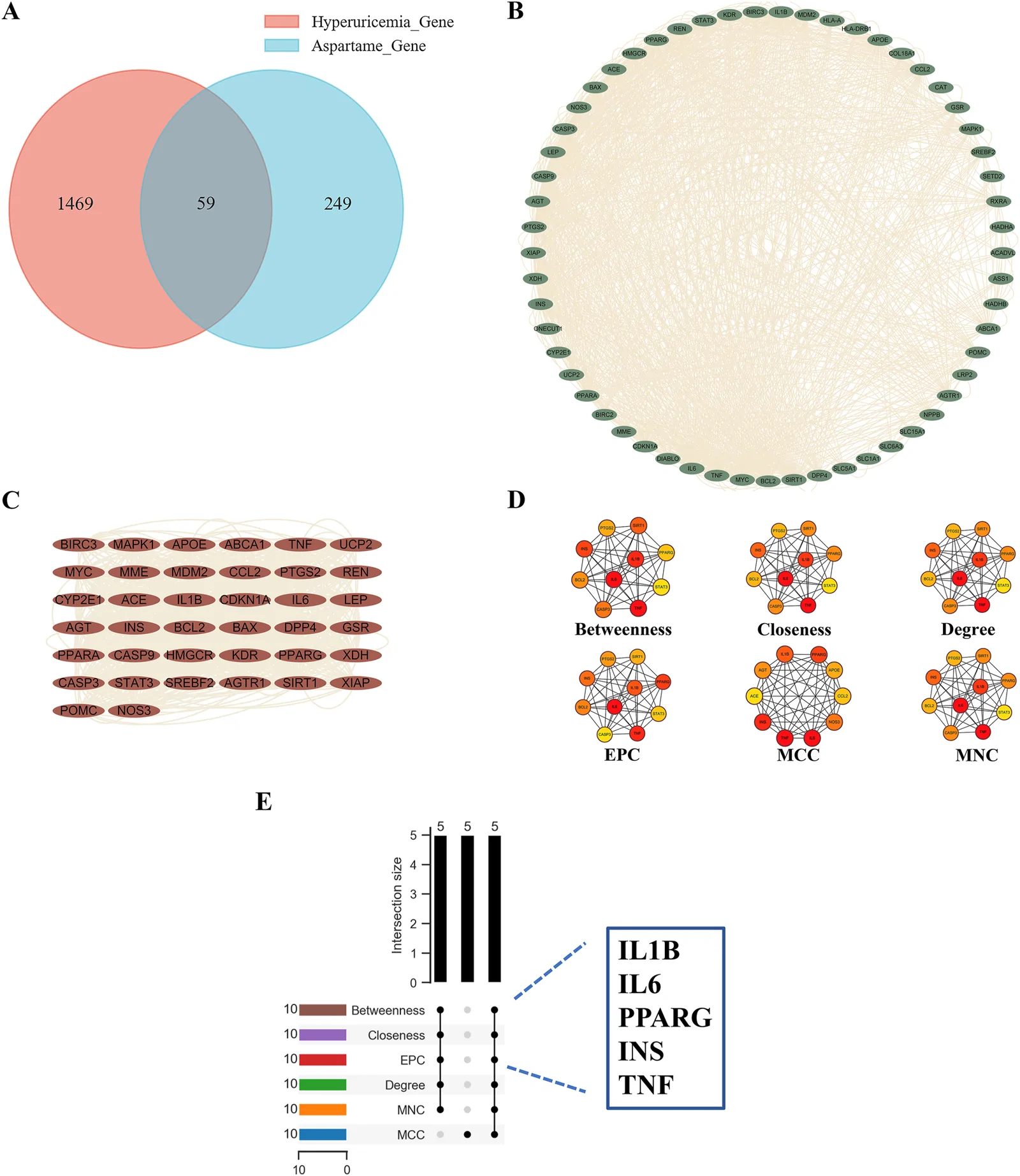
Primary discovery-stage network analysis linking aspartame-associated candidate records with hyperuricemia-associated genes. (A) Intersection between the 308 candidate records and the 1,528 hyperuricemia-associated labels. (B) STRING PPI network of the shared genes. (C) Highest-scoring MCODE module (score=23.568; 38 genes). (D–E) Primary topological hub candidates identified by intersecting the top 10 genes from MCC, MNC, Degree, EPC, Closeness, and Betweenness.

The STRING export contained 57 unique nodes and 1,082 interaction records (Figure 3B). MCODE identified a 38-gene module with the highest score (23.568; Figure 3C). Intersecting the top 10 genes ranked by MCC, MNC, Degree, EPC, Closeness, and Betweenness identified IL1B, IL6, PPARG, INS, and TNF (Figure 3D and E). These genes formed the primary topological hub-candidate set and were carried forward to transcriptomic evaluation and sensitivity analysis.

### Functional enrichment analysis of aspartame-associated genes in hyperuricemia

GO analysis of the 59 shared genes highlighted biological processes related to lipid storage, response to hypoxia, reproductive-system development, lipid localization, and small-molecule metabolism. Enriched molecular functions included receptor–ligand activity, protease binding, amide binding, and nuclear-receptor activity; the principal cellular-component terms included endocytic vesicles, endocytic-vesicle membranes, and brush-border membranes (Figure 4A).

**Figure 4:**
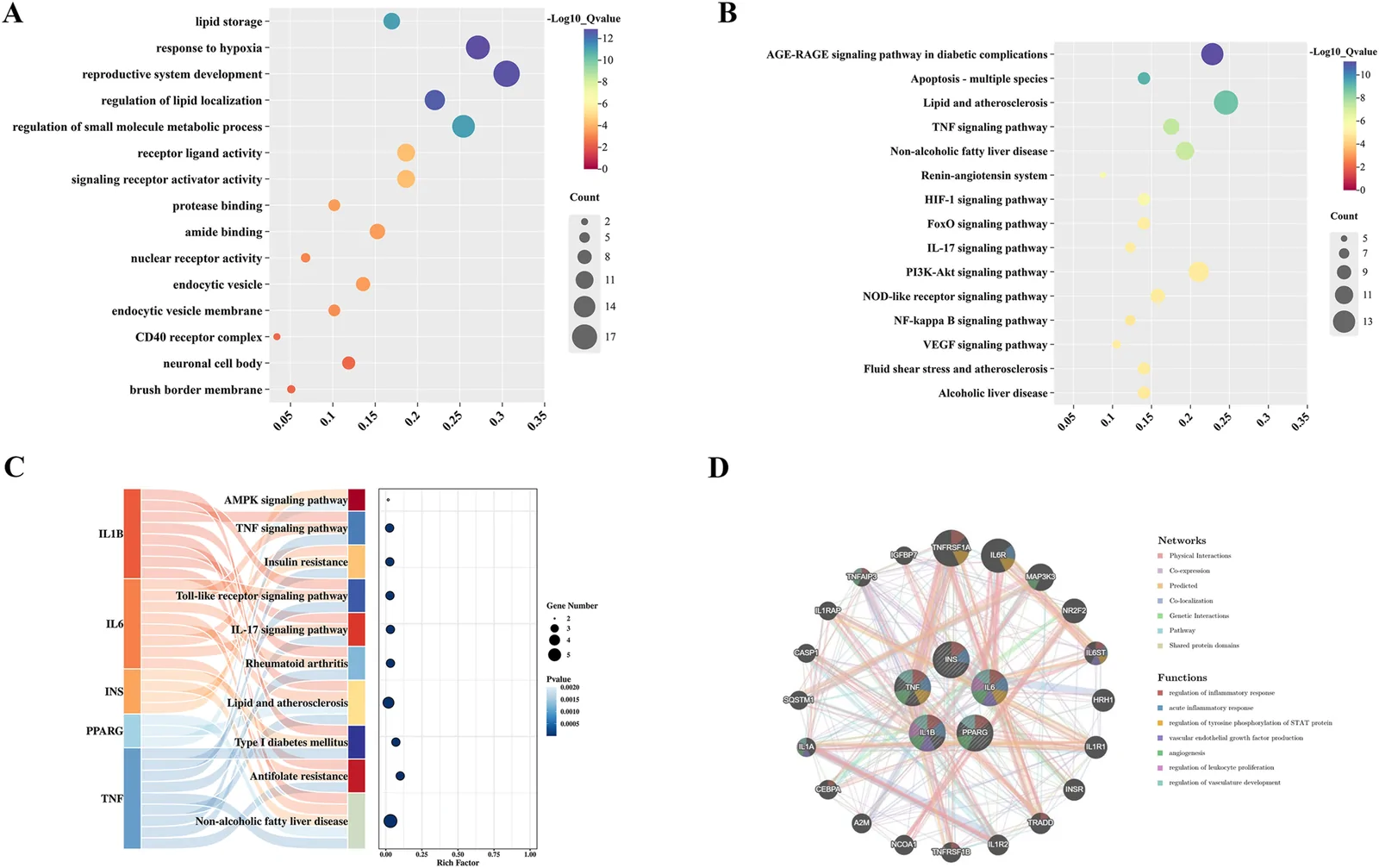
Discovery-stage functional enrichment and interaction analyses. (A) GO enrichment of the 59 shared genes. (B) KEGG enrichment of the primary 59-gene set. (C) Exploratory KEGG analysis of the five primary topological hub candidates. (D) GeneMANIA functional interaction network. These results should be interpreted together with the orthogonal and sensitivity analyses in the Supplementary Materials.

KEGG analysis highlighted AGE–RAGE signaling in diabetic complications, lipid and atherosclerosis, non-alcoholic fatty liver disease, TNF, HIF-1, FoxO, IL-17, PI3K–Akt, NF-κB, and NOD-like receptor signaling (Figure 4B). Exploratory enrichment of the five hub candidates additionally implicated AMPK signaling, TNF signaling, insulin resistance, Toll-like receptor signaling, IL-17 signaling, rheumatoid arthritis, lipid and atherosclerosis, type I diabetes mellitus, non-alcoholic fatty liver disease, and antifolate resistance (Figure 4C). GeneMANIA placed the five candidates within a connected inflammatory, metabolic, immune, and vascular interaction network (Figure 4D). These analyses defined the discovery-stage functional hypotheses assessed in the transcriptomic and sensitivity analyses.

### Molecular docking analysis of aspartame with hyperuricemia-related hub proteins

Docking of intact aspartame against the five proteins prioritized in the primary network produced best-ranked Vina scores from −6.715 to −3.325 kcal/mol (Figure 5 and Table 3). PPARG had the most favorable score (−6.715 kcal/mol), followed by IL1B (−5.416 kcal/mol), IL6 (−5.295 kcal/mol), INS (−3.968 kcal/mol), and TNF (−3.325 kcal/mol). Under a common docking protocol, predicted structural compatibility was therefore more favorable for PPARG, IL1B, and IL6 than for INS or TNF.

**Figure 5:**
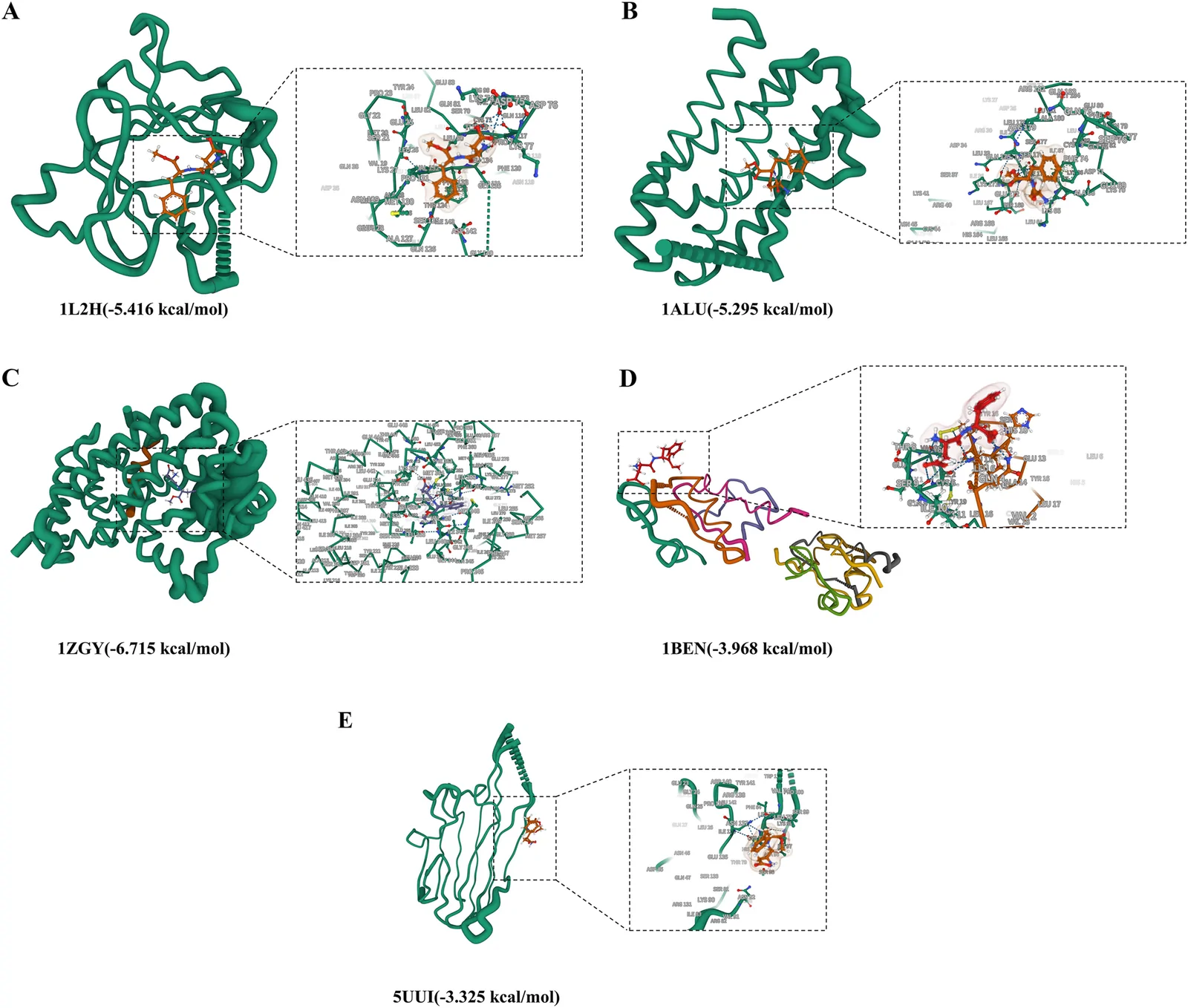
Exploratory docking poses of intact aspartame with proteins selected from the primary network: (A) IL1B, (B) IL6, (C) PPARG, (D) INS, and (E) TNF. Numerical Vina scores are summarized in Table 3. The static poses illustrate predicted structural compatibility and do not constitute experimental binding validation. Only IL1B was retained after CTD-only deletion.

**Table 3:** Molecular docking of aspartame with the core hyperuricemia targets.

Sweetener	Protien	PDB number	Evidence tier	Binding energy, kcal/mol
Aspartame	IL1B	1L2H	Tier 1	−5.416
Aspartame	IL6	1ALU	Tier 2	−5.295
Aspartame	PPARG	1ZGY	Tier 3	−6.715
Aspartame	INS	1BEN	Tier 3	−3.968
Aspartame	TNF	5UUI	Tier 2	−3.325

Vina scores are static docking estimates rather than experimentally measured binding free energies. Evidence tiers were assigned from the integrated transcriptomic and sensitivity analyses; only IL1B was retained after CTD-only deletion. IL1B, Interleukin-1 β; IL6, Interleukin-6; PPARG, Peroxisome proliferator-activated receptor gamma; INS, insulin; TNF, tumor necrosis factor.

In the best-ranked poses, aspartame occupied accessible pockets within the selected protein structures. Close polar contacts occurred near SER289, CYS285, and TYR327 in PPARG; PRO78, THR79, and LYS74 in IL1B; and MET67, SER176, and GLU172 in IL6. Contacts were also observed in the INS and TNF models, although the TNF pose involved CYS77 in the engineered T77C structure, limiting physiological interpretation. These models provide a structural illustration of possible parent-compound–protein compatibility. Because reference-ligand comparison and co-crystal redocking were unavailable, the scores were used only for relative comparison and not as evidence of direct target engagement. Detailed contacts and receptor-specific caveats are provided in Supplementary Table S8.

### Independent human transcriptomic support for the primary network targets

Quality-control analyses of GSE160170 were satisfactory, and principal component analysis showed separation between gout and healthy-control samples (Supplementary Figure S1). Among the five primary hub candidates, IL1B, IL6, and TNF were uniquely mapped and significantly upregulated in gout: IL1B, log2FC=3.091 and FDR=0.0021; IL6, log2FC=2.428 and FDR=0.00094; and TNF, log2FC=2.103 and FDR=0.0047 (Supplementary Figure S2 and Table S2). PPARG and INS were not evaluable because reliable probe identities were unavailable in the annotation.

Within the 59-gene network, PTGS2 (log2FC=2.702, FDR=8.74 × 10^−6^) and VEGFA (log2FC=1.073, FDR=0.0107) were also upregulated, whereas MME was downregulated (log2FC=−1.504, FDR=0.0408). Six network-derived genes therefore met the prespecified differential-expression criteria (Supplementary Figures S3–S4 and Tables S3–S4). Because GSE160170 contains no information on aspartame exposure, these results indicate disease-side biological consistency rather than an exposure-specific response.

### Sensitivity analyses of the primary network and core targets

The first sensitivity analysis examined dependence on CTD-derived records. Removing 171 CTD-only candidates reduced the aspartame-associated set from 308 to 137 records and the hyperuricemia overlap from 59 to 22 genes. The restricted STRING network contained 20 connected nodes and 114 edges. IL1B had the highest degree (13) and betweenness centrality (0.236) and was the only one of the five primary hub candidates retained after CTD-only deletion. TNF, NF-κB, NOD-like receptor, IL-17, AGE–RAGE, and lipid and atherosclerosis signaling remained significant, whereas PI3K–Akt, HIF-1, FoxO, and non-alcoholic fatty liver disease pathways did not.

The second sensitivity analysis assessed disease-side noise in GeneCards. Filtering at the median relevance score, score ≥1.0, the upper quartile, and score ≥2.0 reduced the merged disease sets to 841, 450, 444, and 268 genes, respectively; the corresponding overlaps with the 308 candidate records fell to 35, 26, 23, and 16 genes. Despite this contraction, IL1B, IL6, PPARG, INS, and TNF remained in every filtered overlap and among the top 10 degree-ranked nodes in each restricted network. AGE–RAGE, lipid and atherosclerosis, TNF, IL-17, and NOD-like receptor signaling remained significant at all tested thresholds.

The two analyses refined rather than replaced the discovery network. IL1B and the inflammatory pathway cluster showed the strongest reproducibility across data configurations. IL6 and TNF had independent disease-side support but remained dependent on CTD for exposure-side inclusion. PPARG, INS, and several metabolic or stress-response pathways were more source-dependent and were retained as exploratory findings. Complete gene-level, network, and pathway results are reported in Supplementary Figures S5–S6 and Supplementary Tables S5–S6.

### Relationship of the prioritized findings to urate biology

XDH was the clearest direct urate-production gene in the primary 59-gene network, but it was removed in the CTD-only analysis. The major urate transporters SLC2A9/GLUT9, SLC22A12/URAT1, and ABCG2 were not among the prioritized shared genes. ACE, REN, and NOS3 were therefore interpreted as markers of renal vascular or hemodynamic context, whereas INS and PPARG represented indirect metabolic links to urate handling.

By contrast, IL1B, IL6, TNF, PTGS2, and the TNF/NF-κB/NOD-like receptor/IL-17 pathway cluster predominantly reflected inflammatory responses associated with hyperuricemia or monosodium urate deposition. The prioritized network was therefore more closely aligned with gout-related inflammatory biology than with direct control of urate production or excretion (Supplementary Table S7).

## Discussion

This study combined a broad network-toxicology screen with human transcriptomics and two complementary sensitivity analyses to examine whether aspartame-associated records intersect with hyperuricemia biology. The primary screen identified 308 candidate records, 59 shared genes, and five topological hub candidates. Subsequent analyses narrowed that picture considerably. Across data sources, the most reproducible finding was an IL1B-centered inflammatory signal; support for the remaining hubs and for several metabolic pathways was more conditional. The results therefore define a ranked set of hypotheses rather than a unified five-gene mechanism.

The physicochemical and ADMET predictions provide context for the intact parent molecule, but they do not establish systemic exposure or toxicity. Low predicted membrane permeability and a negligible blood–brain barrier signal are consistent with the highly polar structure of aspartame. More importantly, ingested aspartame is rapidly hydrolyzed in the gastrointestinal tract, and unchanged aspartame is not considered a major circulating species [17], [18], [19]. The high predicted alerts for genotoxicity/Ames activity, hepatotoxicity, nephrotoxicity, and neurotoxicity must likewise be read as model outputs. Their biological relevance depends on model applicability, dose, metabolism, and experimental toxicology [17], 24], 25].

Even within these constraints, the discovery network captured a biologically coherent set of disease-related processes. IL1B, IL6, and TNF mapped to cytokine and innate immune signaling, whereas PPARG and INS mapped to lipid metabolism and insulin signaling. Enrichment of TNF, NF-κB, NOD-like receptor, IL-17, PI3K–Akt, HIF-1, FoxO, AGE–RAGE, and lipid and atherosclerosis pathways is consistent with the intersection of inflammation, metabolic dysfunction, hypoxic stress, and vascular injury in hyperuricemia and gout [3], 13], [26], [27], [28]. Similar network approaches have been applied to aspartame in other disease settings [20], [21], [22]. Here, however, the discovery network was treated as a starting point and then tested against human expression data and alternative database configurations.

GSE160170 provided an independent human disease context. IL1B, IL6, and TNF were upregulated in gout, as were PTGS2 and VEGFA, whereas MME was downregulated. The IL1B result is particularly consistent with established monosodium urate crystal–induced inflammasome activation and cytokine release [12], [14], [15], [16, 28]. PTGS2 added support for an inflammatory effector component, while the meaning of the MME decrease remains uncertain. Because the dataset contains no information on aspartame intake or measured exposure, these findings demonstrate convergence with gout biology, not an exposure-specific response.

The sensitivity analyses were decisive in separating network breadth from evidential robustness. Removing 171 CTD-only candidates reduced the aspartame-associated set from 308 to 137 records and the disease overlap from 59 to 22 genes; only IL1B remained among the five original hubs. In contrast, increasingly stringent GeneCards filters reduced the size of the disease-overlap set but preserved all five hubs among the highly ranked nodes. Weak GeneCards associations therefore broadened the network without fully determining hub selection, whereas CTD had a much larger influence on its exposure-side composition. The two analyses address different questions and should be interpreted together.

Taken together, these results place IL1B at the highest priority. It remained central after CTD-only deletion and was upregulated in GSE160170. PTGS2 and MME also combined source-deletion retention with transcriptomic alteration, although their roles in the proposed exposure–disease relationship are less clear. IL6 and TNF occupy an intermediate tier: both were supported in the human dataset and remained stable under GeneCards filtering, but neither had exposure-side support independent of CTD. VEGFA showed a similar disease-supported, source-dependent pattern. PPARG and INS received the weakest integrated support because both were CTD-dependent and could not be evaluated reliably in GSE160170. Their established roles in metabolic regulation remain relevant [13], 26], but the present data do not support treating them as robust aspartame-associated targets.

The connection to serum urate itself was less direct. XDH was the most explicit urate-production node in the primary network, yet it disappeared after CTD-only deletion [7]. The principal urate transporters SLC2A9/GLUT9, SLC22A12/URAT1, and ABCG2 were not prioritized [3], [4], [5], [6]. ACE, REN, and NOS3 are more plausibly interpreted as indicators of renal hemodynamic or vascular context, whereas INS and PPARG may reflect the metabolic environment that influences urate clearance. By comparison, IL1B, IL6, TNF, PTGS2, and the TNF/NF-κB/NOD-like receptor/IL-17 pathway cluster are closely linked to the inflammatory consequences of urate accumulation and crystal deposition. The reproducible signal thus concerns gout-associated inflammation more strongly than direct regulation of urate production or excretion.

The docking analysis should be viewed in the same measured way. Intact aspartame produced scores from −6.715 to −3.325 kcal/mol under a common AutoDock Vina protocol, with comparatively more favorable values for PPARG, IL1B, and IL6 than for INS or TNF. The best-ranked poses illustrated accessible pockets and close polar contacts, but they did not establish binding efficiency, target engagement, or biological activity. Reference-ligand comparison, co-crystal redocking, and RMSD validation were unavailable, and several receptor structures introduced additional caveats, including engineered IL1B and TNF variants. The favorable PPARG score cannot outweigh the weak integrated evidence for PPARG, while the TNF pose involving engineered CYS77 warrants particular caution.

The recurrence of IL1B, IL6, and TNF is biologically plausible but not evidence of novelty or specificity. These cytokines are established inflammatory hubs and are readily recovered by literature-rich interaction databases and centrality-based algorithms. Their prominence probably reflects both genuine biological importance and dense prior annotation. The contribution of this study lies instead in calibrating the initial network: source-specific evidence was kept distinct, nonevaluable findings were retained, human data provided an independent disease context, and sensitivity analyses separated disease-side stability from exposure-source dependence. This framework identifies which signals deserve experimental priority and which remain database-dependent hypotheses.

Several features strengthen the analysis. Broad retrieval preserved discovery sensitivity, while source annotation prevented computational predictions, target-search records, and curated chemical–gene associations from being treated as equivalent evidence. Human transcriptomics added an independent disease layer, and the two sensitivity analyses tested complementary dimensions of robustness. The study also retained informative negative findings, including the absence of major urate transporters, the inability to evaluate PPARG and INS in GSE160170, and the lack of docking-validation metrics. Together, these features define a realistic translational implication: the results nominate an IL1B-centered inflammatory axis for focused follow-up, but they do not support clinical risk estimation or changes in recommendations on aspartame use.

The study also has important limitations. GSE160170 included only 12 participants, represented primary gout rather than asymptomatic hyperuricemia, and contained no measured aspartame exposure. Database coverage and literature bias may have influenced candidate selection and network topology; SwissTargetPrediction probabilities were unavailable, the ChEMBL export was not compound-specific bioactivity data, and the CTD summary lacked study-level attribution. No disease-matched or degree-matched random-network analysis was performed. The parent compound and its metabolites were not evaluated separately, and the docking workflow lacked prospective controls and experimental confirmation. Future studies should combine measured intake or biomarker-based exposure with longitudinal serum urate, renal urate-handling phenotypes, and inflammatory markers. Dose- and time-resolved experiments should compare intact aspartame with phenylalanine, aspartate, methanol, and relevant downstream metabolites and should test both urate transport and the IL1B-centered inflammatory axis. Such work is needed to determine whether the pattern reported here reflects an exposure-responsive mechanism or the broader inflammatory architecture of gout.

## Conclusions

Taken together, the high-sensitivity network, human transcriptomic data, and source-focused sensitivity analyses converged on an IL1B-centered inflammatory signal. IL6 and TNF were supported in gout but depended on CTD for exposure-side inclusion, whereas PPARG, INS, and several metabolic pathways remained exploratory. Evidence for direct involvement of core urate-transport mechanisms was limited, and docking of intact aspartame should be regarded as an unvalidated structural scenario. The findings identify priorities for targeted experiments but do not establish that aspartame alters serum urate or causes hyperuricemia.

## Supplementary Material

Supplementary Material

Supplementary Material
