# Supplementary material for: Integrative network toxicology and bioinformatics reveal the potential toxicological effects of aspartame on hyperuricemia
Source: Open Med (Wars). 2026 Nov 12;21(1):20261557. doi: 10.1515/med-2026-1557 (PMC13758588; doi:10.1515/med-2026-1557)
Supplement: Supplementary file 1 [file j_med-2026-1557_suppl_002.docx]

**Supplementary Materials**

This file contains the supplementary methods and figures supporting the analyses reported in the manuscript. Complete row-level tables, sample-level data, source annotations, sensitivity-analysis results, and computational provenance are provided in the accompanying Excel file, Supplementary_Tables_and_Data.xlsx.

**Supplementary Methods**

**S1. Database provenance and evidence annotation**

Database searches were documented with their available dates, inputs, raw record counts, standardization procedures and evidence interpretation. SwissTargetPrediction was queried on December 9, 2025, using the canonical SMILES of intact aspartame with Homo sapiens selected as the target species; all 100 returned labels were retained to preserve discovery sensitivity. The available export did not contain prediction probabilities, and no probabilities were reconstructed retrospectively. ChEMBL was queried on December 9, 2025, using the term “aspartame”; 93 target-search rows were exported, 34 were human records, and expansion/deduplication of multi-accession entries yielded 44 unique human UniProt accession or gene labels. Because this was a target-search export rather than an aspartame-specific bioactivity table, no affinity, assay-confidence or pChEMBL threshold was applied. CTD was queried on December 9, 2025, using the specific Aspartame entry (Chemical ID D001218). After blank rows were removed, 178 unique genes remained. Gene ID, Interaction Count and Organism Count were retained as annotations; Interaction Count was treated as a record-count metric rather than a confidence score. The CTD gene-level summary did not provide species identities, supporting PMIDs, interaction actions, exposure formulation or parent-versus-metabolite attribution. GeneCards returned 1,455 hyperuricemia-associated genes, and OMIM contributed 111 records representing 110 unique labels; after removing 37 shared labels, 1,528 disease-associated labels remained. The source-level annotations for the 308 candidate records are provided in Supplementary Tables S1A–S1B. ChEMBL accession mapping is provided in Supplementary Table S1C.

**S2. GSE160170 transcriptomic analysis**

GSE160170 was used as an independent disease-side transcriptomic dataset. The analysis included six primary-gout peripheral blood mononuclear cell samples and six healthy controls; the gout samples consisted of three intercritical and three acute-gout samples. Normalized log2 probe-expression values were used for distribution checks, density plots, principal-component analysis, sample-to-sample correlation analysis and sample-level visualization. The primary differential-expression comparison combined all gout samples and compared them with controls using limma in R. Benjamini–Hochberg-adjusted P < 0.05 and |log2 fold change| > 1 were prespecified as differential-expression criteria. The five primary hub candidates and the 59 network-derived genes were queried against the available validated probe-to-gene mapping. IL1B, IL6 and TNF had uniquely mapped representative probes and were evaluated at sample level. Reliable probe identities could not be assigned to PPARG or INS from the available significant-probe annotation; these genes were therefore reported as not evaluable rather than nonsignificant. Comparisons among control, intercritical-gout and acute-gout groups were considered exploratory because each gout subgroup contained only three samples. Results are provided in Supplementary Figures S1–S4 and Supplementary Tables S2–S4; sample-level data are provided as Supplementary Data S1–S2.

**S3. CTD-only source-deletion analysis**

CTD-only genes were defined as genes present in CTD but absent from both SwissTargetPrediction and ChEMBL. These genes were removed, and the retained exposure-side candidate set was re-intersected with the original hyperuricemia-associated gene set. This reduced the aspartame-associated candidate set from 308 to 137 records and the disease-overlap set from 59 to 22 genes. The original STRING edge table was restricted to retained genes, and degree, betweenness centrality and closeness centrality were recalculated with NetworkX version 3.6.1. For pathway-level assessment, the original 59-gene set and the CTD-only deletion set were analyzed with the same KEGG human gene-to-pathway mapping retrieved on August 1, 2026. Hypergeometric over-representation tests used 9,421 KEGG pathway-linked human genes as the background and evaluated 372 pathways. P values were adjusted using the Benjamini–Hochberg method; BH-FDR < 0.05 was considered significant. Full gene-level, network and pathway results are provided in Supplementary Figure S5 and Supplementary Tables S5A–S5F.

**S4. GeneCards filtering analysis**

Disease-side robustness was assessed by retaining all OMIM records and applying four progressively stricter GeneCards relevance-score thresholds: the observed median score (0.690937), score ≥ 1.0, the upper-quartile score (1.074029) and score ≥ 2.0. For each threshold, the merged disease-gene set, candidate–disease overlap, restricted STRING network, degree ranking and KEGG enrichment were recalculated under identical analytical settings. These thresholds were used to evaluate robustness to disease-gene filtering and were not interpreted as definitive biological cutoffs. Complete results are provided in Supplementary Figure S6 and Supplementary Tables S6A–S6E.

**S5. Evidence integration and targeted urate-mechanism classification**

Findings were integrated in three evidence tiers. Tier 1 comprised genes or pathways retained after CTD-only deletion and, where evaluable, supported in GSE160170. Tier 2 comprised genes supported in the human dataset but dependent on CTD for exposure-side inclusion. Tier 3 comprised CTD-dependent genes without independent transcriptomic support and pathways not retained after source deletion. A targeted literature-based classification separated direct urate production, core urate transport, indirect renal or metabolic context and downstream gout inflammation. These categories guided interpretation but did not constitute a quantitative confidence score. Definitions and gene/pathway assignments are provided in Supplementary Table S7.

**S6. Docking transparency**

Docking used intact aspartame as the standardized parent-compound ligand. The receptor structures were IL1B (1L2H), IL6 (1ALU), PPARG (1ZGY), INS (1BEN) and TNF (5UUI). The available preparation records documented removal of water molecules, addition of polar hydrogens and conversion of receptor and ligand structures to PDBQT format. AutoDock Vina 1.2.2 was used, with an archived search box of 30 Å × 30 Å × 30 Å and a reported grid-point distance of 0.05 nm. Exact grid centers, exhaustiveness, random seeds, number of output modes, energy range and raw Vina logs were not recoverable. Co-crystal ligand redocking, positive-control docking and RMSD validation were not performed. Docking scores are therefore reported in kcal/mol and interpreted only as exploratory estimates of static structural compatibility. Residues within 4 Å of the docked ligand and close polar-atom contacts within 3.5 Å were summarized. Receptor-specific caveats and the complete parameter audit are provided in Supplementary Tables S8A–S8B.

**Supplementary Figures**

Supplementary Figure S1A. Distribution of normalized log2 expression values across the 12 GSE160170 samples.

Supplementary Figure S1B. Density distributions of normalized log2 expression values across the 12 samples.

Supplementary Figure S1C. Principal-component analysis comparing six patients with primary gout and six healthy controls.

Supplementary Figure S1D. Principal-component analysis showing controls, intercritical gout and acute gout.

Supplementary Figure S1E. Sample-to-sample correlation heatmap for normalized expression.

Supplementary Figure S2. Sample-level expression of IL1B, IL6 and TNF. PPARG and INS were not plotted because reliable probe identities were unavailable.

 

Supplementary Figure S3. Row-standardized expression heatmap of IL1B, IL6, TNF, PTGS2, VEGFA and MME.

Supplementary Figure S4. Gout-versus-control limma effects and BH-adjusted P values for the six supported network genes.

Supplementary Figure S5A. Proportions retained after removal of CTD-only genes.

Supplementary Figure S5B. Comparison of KEGG pathway significance before and after CTD-only removal.

Supplementary Figure S5C. Degree ranking in the induced non-CTD-only PPI network.

 

Supplementary Figure S6A. Candidate–disease overlap counts across GeneCards filters.

Supplementary Figure S6B. Stability of key KEGG pathways across GeneCards filters.

**Supplementary figure legends**

Supplementary Figure S1A. Distribution of normalized log2 expression values across the 12 GSE160170 samples.

Supplementary Figure S1B. Density distributions of normalized log2 expression values across the 12 samples.

Supplementary Figure S1C. Principal-component analysis comparing six patients with primary gout and six healthy controls.

Supplementary Figure S1D. Principal-component analysis showing controls, intercritical gout and acute gout.

Supplementary Figure S1E. Sample-to-sample correlation heatmap for normalized expression.

Supplementary Figure S2. Sample-level expression of IL1B, IL6 and TNF. PPARG and INS were not plotted because reliable probe identities were unavailable.

Supplementary Figure S3. Row-standardized expression heatmap of IL1B, IL6, TNF, PTGS2, VEGFA and MME.

Supplementary Figure S4. Gout-versus-control limma effects and BH-adjusted P values for the six supported network genes.

Supplementary Figure S5A. Proportions retained after removal of CTD-only genes.

Supplementary Figure S5B. Comparison of KEGG pathway significance before and after CTD-only removal.

Supplementary Figure S5C. Degree ranking in the induced non-CTD-only PPI network.

Supplementary Figure S6A. Candidate–disease overlap counts across GeneCards filters.

Supplementary Figure S6B. Stability of key KEGG pathways across GeneCards filters.

Complete row-level Tables S1A–S8B, Supplementary Data S1–S2, supporting maps and computational provenance are provided in Supplementary_Tables_and_Data.xlsx.
